# Hydrogel as a Platform for Point-of-Care Calcium Determination in Blood

**DOI:** 10.3390/gels12010028

**Published:** 2025-12-29

**Authors:** Tatiana N. Tikhonova, Anastasia V. Barkovaya, Yuri M. Efremov, Vladimir I. Panov, Peter S. Timashev, Victor V. Fadeev

**Affiliations:** 1 Department of Physics, M.V. Lomonosov Moscow State University, Leninskie Gory 1/2, 119991 Moscow, Russia; anastasia.bark18@gmail.com (A.V.B.); panov@spmlab.phys.msu.ru (V.I.P.); vfadeev@physics.msu.ru (V.V.F.); 2 Institute for Regenerative Medicine, Sechenov University, 8-2 Trubetskaya St., 119991 Moscow, Russia; yu.efremov@gmail.com (Y.M.E.); timashev.peter@gmail.com (P.S.T.); 3 Department of Chemistry, M.V. Lomonosov Moscow State University, Leninskie Gory 1/2, 119991 Moscow, Russia; 4 World-Class Research Center “Digital Biodesign and Personalized Healthcare”, Sechenov First Moscow State Medical University 8-2, Trubetskaya St., 119991 Moscow, Russia

**Keywords:** hydrogel, gelatin, calcium concentration in plasma, arsenazo III, point of care, fluorescent sensor

## Abstract

Calcium is a key macroelement involved in a range of physiological processes in the body, and its concentration in blood is an important diagnostic indicator in various diseases. This work presents a novel rapid method for the point-of-care determination of total calcium content in patient blood, by applying a drop of capillary blood from a finger onto a hydrogel. Gelatin hydrogel, modified with an optical sensor for calcium, Arsenazo III, was used as a platform for the separation of blood into plasma and erythrocytes. A comparative analysis of various types of hydrogel materials (polyacrylamide, PVA, Fmoc-FF, carbomer, carbopol, gelatin) was performed, demonstrating that among the studied systems, only gelatin hydrogel is suitable as a platform for the determination of calcium in blood plasma. The binding of calcium ions from blood plasma with the calcium sensor embedded in the hydrogel leads to a change in the absorption spectrum of the system, enabling photometric determination of calcium concentrations below and above the normal range in blood plasma. Therefore, this rapid assay allows monitoring of calcium metabolism disorders in the human organism. The method is characterized by its speed, simplicity of sample preparation, and potential for integration into clinical practice.

## 1. Introduction

Biomaterials are widely used in various fields of biomedicine and continue to be actively studied due to their enormous diversity and great potential for practical application [1,2,3,4,5]. Among the different types of biomaterials are hydrogels, three-dimensional structures formed from synthetic or natural polymers capable of absorbing and retaining significant amounts of water (70–95%) [6]. Due to their porous structure, biocompatibility, biodegradability, and similarity to the extracellular matrix, hydrogels are particularly attractive for various medical applications, including tissue engineering, tissue regeneration, controlled release of therapeutic agents (proteins, drugs), contact lenses, wound dressings, sensors, and more [7,8,9,10].

Depending on the specific medical application, hydrogels are required to meet different criteria: for transplantation and regenerative technologies, mechanical strength and biodegradability are important. For example, by varying the stiffness of biomaterials, different tissue types can be mimicked—from brain tissue (0.1–1 kPa) to endothelial (1–3 kPa) and muscle (15–20 kPa) tissue [11,12,13,14]. For drug delivery systems, controlled porosity and the ability to release active substances due to swelling or compression of the hydrogel and diffusion of the drug through the polymer network are the key properties [15]. For sensor applications, optical transparency, chemical inertness, and the ability to incorporate analytical probes are required [16].

Currently, one of the relevant directions in biomedicine is the development of methods for determining various analytes in blood in a point-of-care format. In particular, the determination of calcium ion concentration in patient blood is an important task, as calcium level is a crucial diagnostic indicator for assessing bone tissue status, parathyroid function, and the cardiovascular and nervous systems [17,18]. Traditionally, calcium determination is carried out in laboratory settings, requiring blood sampling and subsequent laboratory analysis. There are two main laboratory methods for determining calcium in blood plasma: the colorimetric method for total calcium analysis and the potentiometric method using an ion-selective electrode for ionized calcium determination [19].

Methods based on the analysis of saliva and sweat, where calcium concentration may correlate with that in blood plasma (albeit with certain limitations), are also being actively developed. Both colorimetric rapid tests using reagents that change color in the presence of calcium and biosensors for saliva and sweat based on electrochemical or optical detection are used. These provide continuous monitoring but require consideration of factors affecting the composition of biological fluids (hydration, diet, physical activity) [20,21].

**Figure 1:**
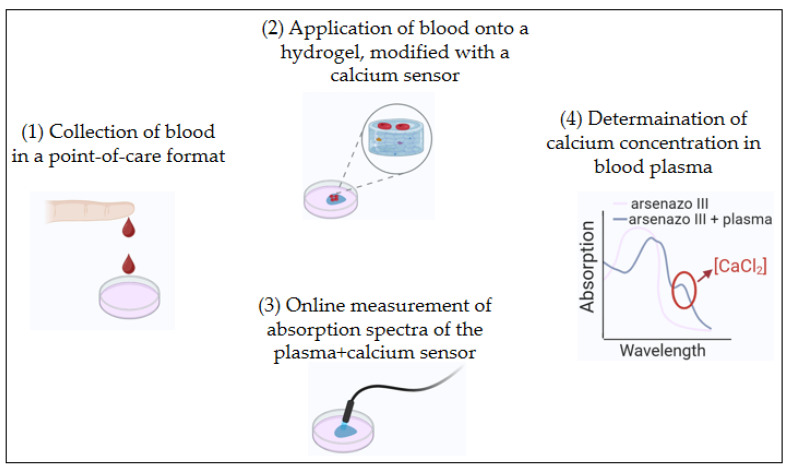
Schematic of the rapid point-of-care assay for calcium concentration in blood. (1) A drop of blood is applied to the hydrogel surface. (2) Plasma penetrates into the gel, while erythrocytes remain on its surface. (3) Inside the hydrogel the calcium probe—Arsenazo III—is incorporated, which is capable of selectively binding calcium ions. Online measurement of absorption spectra of plasma + Arsenazo III is carried out. (4) Determination of calcium concentration of blood plasma using absorption spectra.

Here, we propose a method for determining calcium in blood in a point-of-care format, with blood sampling performed from a finger prick. Hydrogel serves as a platform for separating blood into plasma and erythrocytes (and other cells): a drop of blood is applied to the hydrogel surface, and then plasma penetrates into the gel, while erythrocytes remain on its surface. Inside the hydrogel the calcium probe—Arsenazo III—is incorporated, which is capable of selectively binding calcium ions. Upon binding of the calcium sensor to calcium ions, its optical properties change—this fact can be detected photometrically: the absorption spectrum of Arsenazo III is measured and the calcium concentration in the patient’s blood is determined (see Figure 1).

The selection and modification of hydrogel properties (pore size, gel permeability for plasma, interaction with the calcium sensor) allow for rapid separation of whole blood into plasma and erythrocytes, with the latter remaining on the gel surface. The measurement accuracy allows the detection of hypocalcemia and hypercalcemia, i.e., monitoring of calcium metabolism disorders in blood. This approach thus combines ease of use and the possibility of rapid analysis in a point-of-care format.

## 2. Results and Discussion

### 2.1. Selection of Gel for Determination of Calcium Ion Concentration in the Gel + Arsenazo III System

Hydrogel was chosen as the platform for applying drops of plasma or whole blood, as it is a convenient polymer material whose properties can be tuned by changing gel concentration or crosslinking, thus adjusting pore size [22]. In the first stage, the ability of plasma to penetrate the gel and the possibility of determining its concentration by measuring changes in the optical properties of the incorporated molecular sensor were analyzed. Detailed photographs of the hydrogels, showing all stages of application of the blood samples under investigation and the color changes in the gels resulting from the binding of Arsenazo III with calcium ions, are presented in Figure S1.

Very small amounts of plasma can be applied to the gel: in experiments, 50 μL was used, but even smaller volumes (20–50 μL) are feasible, making it a convenient research platform. Both calibrator (calcium chloride solution of known concentration) and patient blood plasma were applied to the gel. It was necessary that the gel stiffness and pore size allowed free passage of calcium ions and calcium-bound plasma proteins into the gel. The ratio of calcium probe concentration was also optimized for accurate measurement of calcium ions in plasma to detect hypocalcemia and hypercalcemia, i.e., to monitor calcium metabolism disorders in blood.

The second stage of the experiment involved applying whole blood to the gel in such a way that erythrocytes would remain on the gel surface, while plasma and calcium ions would penetrate the gel. This was necessary because the absorption peaks of hemoglobin (λ = 550, 580 nm) [23], present in erythrocytes, are close to the absorption peak of the Arsenazo III + CaCl_2_ complex (λ = 650 nm) [24], and high erythrocyte content in plasma would result in a significant background, making the use of optical impossible.

Arsenazo III, a widely used organic reagent for the spectrophotometric determination of calcium ions and other divalent cations in biological and clinical samples [25], was used as the sensor. Therefore, another requirement for the hydrogel was that it should not contain substances capable of binding to Arsenazo III in the absence of calibrator/blood plasma.

Various polymers were investigated as hydrogels: polyacrylamide gel (PAAG), self-assembling Fmoc-FF peptide gel, PVA gel (from polyvinyl alcohol), carbomer gel, and gelatin gel. All these gels form rapidly, they are widely used in regenerative medicine, and their properties such as assembly, structure, pore size, stiffness, etc., are well studied [26,27,28,29,30]. However, only gelatin gel was suitable for our task.

Polyacrylamide gel, PVA gel, and carbomer 2020 contained substances that, in the absence of calcium ions, bound Arsenazo III, resulting in an absorption spectrum with a peak corresponding to calcium binding, making it impossible to determine which fraction of Arsenazo III remained free and which fraction was already bound to gel components before adding calcium-containing liquid to the hydrogel. Absorption spectra of PAAG + Arsenazo III, PVA + Arsenazo III, and carbomer + Arsenazo III systems are shown in Figure S2. The Fmoc-FF peptide gel was unsuitable because it was too soft, with fibers spaced far apart [31], allowing both calcium ions and erythrocytes (containing hemoglobin, whose absorption spectrum significantly overlaps with that of the Arsenazo III + CaCl_2_ system; see Figure S2) to penetrate the gel when whole blood was applied.

**Figure 2:**
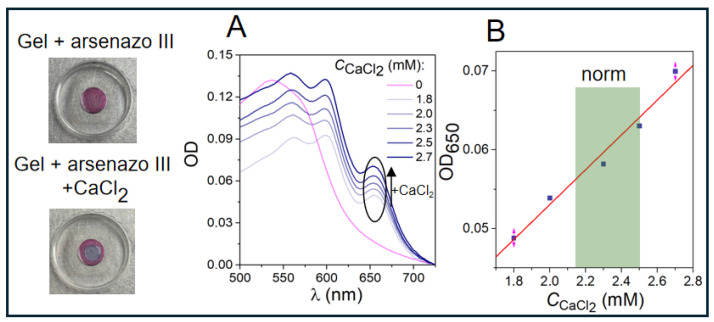
(**A**) Absorption spectrum of pure Arsenazo III in gelatin hydrogel (pink curve) and after addition of calcium ions (blue curves). (**B**) Optical density at λ = 650 nm as a function of calcium concentration in the hydrogel. The green zone in the figure indicates the normal limits for calcium ion levels in blood plasma. The pink arrows indicate the standard deviation in the experimental data.

After studying the interaction of all tested gels with the calcium probe Arsenazo III, gelatin gel was found to be well suited for our application. In this system, the gel is pink in color (see upper photo in Figure 2). When a calcium-containing liquid is applied, the probe color changes to blue (see lower photo in Figure 2), and its absorption spectrum features several lines corresponding to the formation of the Arsenazo III + Ca complex (see Figure 2A, pink and blue curves, respectively). The absorption spectrum of pure Arsenazo III in gelatin hydrogel is characterized by a maximum at 535 nm, while the spectrum of the Arsenazo III + calcium system has three maxima at 560, 600, and 650 nm, indicating a red shift in the Arsenazo III absorption maximum and formation of calcium–Arsenazo III complexes. Normalized values of these peaks at various calcium concentrations are shown in Figure S4. All points fall on a straight line, indicating that the behavior of their intensities is similar. All three peaks describe the process of calcium binding to the dye in the gel. The absorption peak at 650 nm is commonly used as the main indicator for the Arsenazo III + CaCl_2_ complex [25] and thus will be the focus of further analysis. Figure 2B shows the change in optical density of Arsenazo III in gelatin gel at 650 nm upon addition of calcium concentrations typical for healthy human plasma (*C*_CaCl2_ = 2.15–2.50 mM).

This dependence is linear, indicating that gelatin gel with embedded Arsenazo III can be used for accurate determination of calcium concentration in test liquids applied to the hydrogel.

### 2.2. Determination of Calcium Ion Concentration in Patient Blood Plasma Applied to Hydrogel

**Figure 3:**
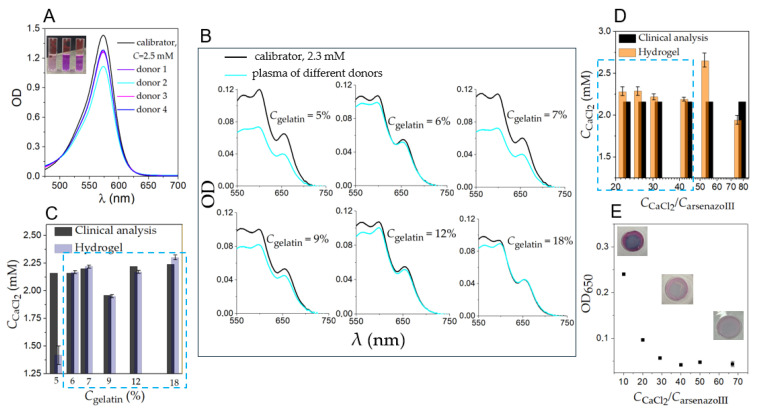
(**A**) Absorption spectra for blood plasma in calcium-olvex reagents for different donors. The photo on the left shows images of the samples (left to right): calcium-olvex reagents, reagents with added calibrator (a solution of calcium chloride at a concentration of 2.5 mM), and calcium-olvex reagents with added patient’s plasma. (**B**) Absorption spectra for blood plasma from different patients (blue curves) and for the calibrator (black curves). In these and all subsequent experiments with hydrogels, the calibrator was a solution of calcium chloride with a concentration of *C*_calib_ = 2.3 mM. The graphs are presented for hydrogels with varying gelatin concentrations: *C*_gelatin_ = 5–18%. *C*_arsenazoIII_ = const = 7 × 10^−5^ M. (**C**) Comparison of calcium concentration values in donor blood plasma, assessed using calcium-olvex (colorimetric method, in solution)—labeled as clinical analysis (black bars) and blood plasma applied to hydrogel (purple bars), considering hydrogels with different gelatin concentrations. (**D**) Comparison of calcium concentration values in donor blood plasma, assessed using calcium-olvex (colorimetric method, in solution)—labeled as clinical analysis (black bars) and blood plasma applied to hydrogel (orange bars), considering hydrogels with varying ratios of calcium to Arsenazo III concentration. (**E**) Dependence of optical density at wavelength λ = 650 nm on the ratio of calcium concentration to Arsenazo III concentration. Above each data point are photographs of samples with the corresponding *C*_CaCl2_/*C*_ArsenazoIII_ ratio. The blue frames highlight areas where clinical analysis data are in good agreement with experimental data.

The application of the hydrogel + Arsenazo III system for accurate determination of calcium concentration in plasma applied to the hydrogel was further investigated. Initially, calcium concentration in donor plasma samples was determined using the “calcium-olvex” kit, intended for quantitative determination of calcium in human serum or plasma by colorimetric method in clinical laboratory diagnostics. On the left in Figure 3A is a photo of samples (left to right): calcium-olvex reagents, reagents with calibrator added (calcium chloride solution at 2.5 mM), and calcium-olveks reagents with patient plasma added. The calcium concentration in patient blood plasma was calculated by the following formula:

(1) $$C_{donor}=\frac{D_{donor}}{D_{calibrator}}\cdot C_{calibrator}$$

To select a hydrogel with the desired properties—such that the calcium from plasma applied to the hydrogel was present in the hydrogel at the same concentration as that in the patient’s blood plasma—both the concentration of the peptide forming the hydrogel and the concentration of Arsenazo III incorporated into the gel were varied. Initially, gels with different gelatin concentrations were studied, with the range *C*_gelatin_ = 5–18%. Absorption spectra for blood plasma from different patients (blue curves) and for the calibrator (black curves) are shown in Figure 3B. In these and all subsequent experiments with hydrogels, the calibrator was a calcium chloride solution with a concentration of *C*_calib_ = 2.3 mM. The graphs for each gelatin concentration in the gel show calcium concentrations in patient blood plasma as determined by clinical analysis (calcium-Olvex) and in blood plasma that had passed into the hydrogel. The calcium concentration in plasma that penetrated the hydrogel was calculated using Formula (1): the value for *D*_donor_ was taken as λ = 650 nm from the blue curves in Figure 3B, and the value for *D*_calib_ was taken as λ = 650 nm from the black curves in Figure 3B.

Figure 3C presents a comparison of calcium values in blood plasma samples obtained by clinical analysis and via hydrogel. It can be seen that for gels with gelatin concentrations *C*_gelatin_ = 6–9%, the calcium values determined by both methods were in good agreement. For gels with *C*_gelatin_ = 12–18%, the calcium concentration in the gel differed slightly from that in plasma determined by clinical analysis. For example, at *C*_gelatin_ = 12%, the calcium concentration in the gel was *C*_CaCl2_gel_ = 2.17 mM, while in plasma it was *C*_CaCl2_clin.anal_ = 2.22 mM. In the gel with *C*_gelatin_ = 5%, significant differences were observed: *C*_CaCl2_gel_ = 1.42 mM and *C*_CaCl2_clin.anal_ = 2.16 mM. This is explained by the fact that the gel at this concentration was very soft, its structure was unstable, and when plasma was applied, the upper layers of the gel were washed away, leading to non-reproducible experiments as varying volumes of plasma penetrated the hydrogel in repeated trials.

The influence of probe concentration on calcium determination in gel samples was also studied. For this, all gels were made with a single gelatin concentration, *C*_gelatin_ = 7%, and the Arsenazo III concentration was varied from 3 × 10^−5^ M to 2 × 10^−4^ M. Plasma from a single donor with *C*_CaCl2_clin.anal_ = 2.16 × 10^−3^ M was applied to all gels. Good agreement in calcium concentration data was observed for gels where the ratio of calcium to probe concentration was in the range 10–40, corresponding to Arsenazo III concentrations of 2 × 10^−4^ M–6 × 10^−5^ M. This is because at high calcium-to-Arsenazo III ratios (50–70, see Figure 3D), the concentration of calcium-Arsenazo III complexes was too low, and the optical density of Arsenazo III in the gel at 650 nm was insensitive to them (see Figure 3E).

### 2.3. Determination of Calcium Concentration in Patient Blood Plasma and Whole Blood Using the Hydrogel Platform

An experiment was conducted to address whether the hydrogels can be used to determine calcium ion concentration in patient blood plasma if whole blood, rather than plasma, is applied. For this, the hydrogel must have a structure such that erythrocytes remain on the surface, while calcium ions penetrate into the gel.

**Figure 4:**
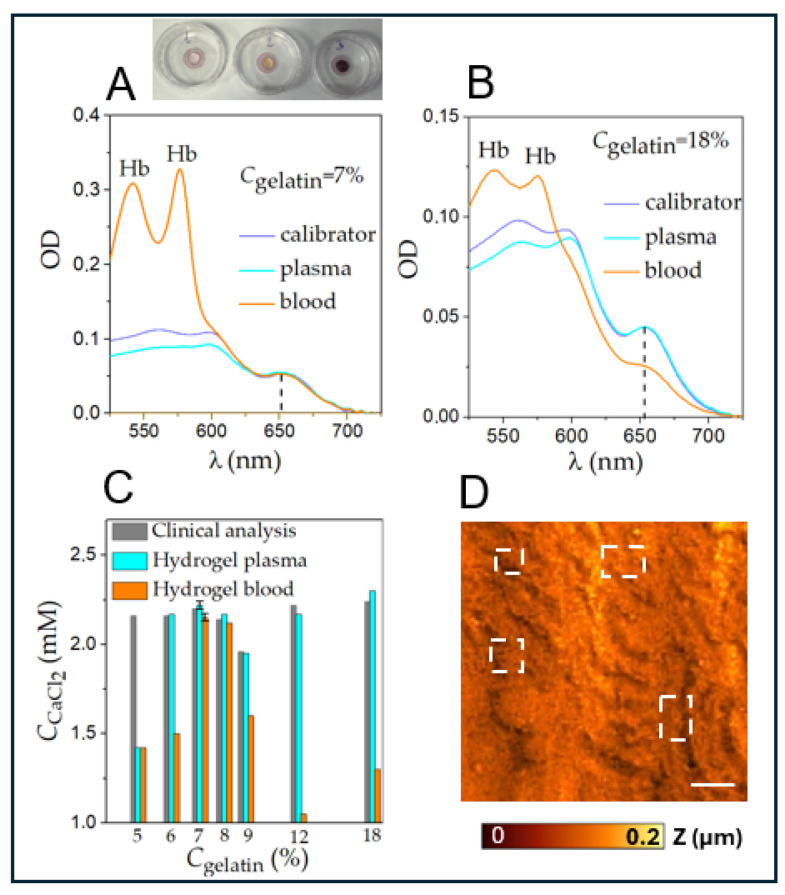
(**A**) Absorption spectra for calibrator (black curve), blood plasma (blue curve), and whole blood (red curve) applied to gelatin gel with *C*_arsenazoIII_ = 6 × 10^−5^ M and (**A**) *C*_gelatin_ = 7% or (**B**) *C*_gelatin_ = 18%. Photos of the applied drops are shown above graph 4(A): 1—calibrator; 2—plasma; 3—whole blood. (**C**) Comparison of calcium concentration values in donor blood plasma, assessed using calcium-Olvex (colorimetric method, in solution)—labeled as clinical analysis (black bars), plasma applied to hydrogel (blue bars), and whole blood applied to hydrogel (red bars), considering hydrogels with different gelatin concentrations. *C*_arsenazoIII_ = const = 7 × 10^−5^ M. (**D**) Topography image of gelatin gel with *C*_gelatin_ = 7%, obtained by scanning ion conductance microscopy. Capillary radius was *R*_tip_ = 50 nm. The pores in the gel are outlined in white frames.

On the same gel, e.g., with the parameters *C*_gelatin_ = 7% and *C*_arsenazoIII_ = 6 × 10^−5^ M, the calibrator (*C*_calib_ = 2.3 mM), plasma, and whole blood from one patient were applied, and it was investigated whether the same calcium concentration passed from whole blood and from plasma (see Figure 4A).

In Figure 4A, it can be seen that for the 650 nm peak, the graphs for the calibrator, plasma, and whole blood coincide, while in the 520–600 nm range, two peaks appear at λ = 545 nm and λ = 575 nm, corresponding to the presence of hemoglobin in the samples. It should be noted that blood remaining on the gel surface was removed before measurement, so the peaks at λ = 545 nm and λ = 575 nm correspond to hemoglobin from lysed erythrocytes entering the gel.

The hemoglobin spectrum does not overlap with the 650 nm peak and does not distort the maximum value of this peak. Figure 4B shows a similar graph but with *C*_gelatin_ = 18%. Although the spectra for calibrator and plasma coincide, the absorption spectrum for whole blood is significantly lower. With increasing peptide concentration in hydrogels, pore size decreased. This explains why, at higher gelatin concentrations, plasma and its contents penetrated the gel less effectively. Figure 4D shows the topography of gelatin gel with *C*_gelatin_ = 7%, obtained by scanning ion conductance microscopy. The capillary radius was *R*_tip_ = 50 nm. For gels with parameters suitable for our purpose (see Figure 4C, marked with red dashed line), i.e., gels with *C*_gelatin_ = 7, 8%, pore size was 300–600 nm.

### 2.4. Standardization of the Method and Robustness Testing

To ensure the practical applicability and reproducibility of the developed assay for calcium determination in whole blood and plasma, we conducted a comprehensive series of robustness and standardization experiments. These addressed potential sources of variability, including differences in gel composition, storage conditions, environmental factors, and sample handling.

#### 2.4.1. Gel Source and Purity

The purity and origin of the gelatin gel were found to be critical factors affecting assay performance. We compared (i) food-grade gelatin from various manufacturers, (ii) gelatin from bovine skin (Sigma-Aldrich, St. Louis, MO, USA, G9382), and (iii) gelatin from bovine skin (Sigma-Aldrich, G9391). Absorption spectra (Figure S5) revealed that only the Sigma-Aldrich gelatins were free of impurities that could interact with the Arsenazo III probe; food-grade gelatins consistently contained divalent metal impurities (notably Ca^2+^), resulting in a detectable peak at 650 nm even in the absence of added calcium. These impurities could confound calcium quantification and are thus unsuitable for assay development. Accordingly, only high-purity, laboratory-grade gelatin is recommended for reproducible results.

#### 2.4.2. Effect of Gel Storage and Thawing

We assessed the impact of storage and thawing of the gelatin gel + Arsenazo III system on assay performance. Gels were stored at −20 °C for 1, 3, or 5 days, then thawed at room temperature for varying durations (5, 10, or 20 min) prior to sample application. Absorption spectra (Figures S6 and S7) demonstrated that a minimum thawing time of 10 min was necessary to ensure consistent diffusion of calcium into the gel and reproducible results. Importantly, storage duration (up to 5 days at −20 °C) did not affect assay outcomes, indicating good stability of the prepared gels under these conditions.

#### 2.4.3. Standardization of Blood Cell Removal and Hemolysis Assessment

The removal of surface blood cells prior to detection is a manual step that can introduce variability. To evaluate its effect, we systematically varied the volume of distilled water used for washing (5, 10, or 15 mL) after whole-blood incubation on the gel. The resulting absorption spectra (Figure S8) showed that while hemoglobin-related peaks at 540 and 575 nm decreased with increased washing, the optical density at 650 nm—used for calcium quantification—remained unaffected. For further validation, we compared the spectra from whole blood and plasma samples from the same donor, confirming that the 650 nm peak was identical in both cases.

To address potential interference from hemolysis, we optimized the gelatin concentration such that only minimal hemoglobin diffused into the gel, thereby preventing distortion of the absorption spectrum near 650 nm. These findings indicate that, under the optimized protocol, the assay is robust to minor variations in washing and is not significantly affected by hemoglobin contamination.

#### 2.4.4. Recommendations for Standardized Operation

Based on these robustness studies, we recommend the following standardized protocol for reliable calcium determination: (1) Use only high-purity, laboratory-grade gelatin. (2) Prepared gels with Arsenazo III should be stored at –20 °C (3) Thaw gels for at least 10 min at room temperature before sample application. (4) After whole-blood application and incubation for 10 min, remove surface blood cells by washing with at least 5 mL of distilled water. (5) Adhere to the optimized gelatin and Arsenazo III concentrations (7–8% gelatin, ~7 × 10^−5^ M Arsenazo III).

These measures ensure the robustness, reproducibility, and practical applicability of the assay for point-of-care calcium determination in blood samples.

### 2.5. Results Discussion

Calcium is one of the most important macroelements, playing a key role in maintaining physiological functions, including blood coagulation, nerve impulse transmission, muscle contraction, and bone tissue maintenance. Measurement of calcium concentration in blood is of significant diagnostic value for a number of diseases such as metabolic disorders, kidney disease, bone system diseases, and parathyroid disorders [32,33].

Currently, various methods exist for determining calcium in blood plasma, differing in principle, accuracy, and accessibility. In this work, a method was developed for rapid analysis of total calcium content in patient blood plasma. The experimental sequence is shown in Figure 1. It is assumed that capillary blood taken from a finger will be applied to a hydrogel containing a calcium probe, Arsenazo III. After 10 min, the liquid that has not penetrated the gel is removed, followed by measurement of absorption spectra from the Arsenazo III + calcium system and determination of calcium concentration in the patient’s blood.

Currently, Arsenazo III is widely used in medical laboratories to determine calcium concentration in human blood plasma, serum, and urine [34,35,36]. It should be noted that this probe binds not only to calcium ions in plasma, but also to magnesium and other divalent ions.

Spectroscopic studies have shown that Arsenazo III exhibits higher selectivity for calcium compared to magnesium; the complex formation constant for Arsenazo III–calcium is an order of magnitude higher than that for Arsenazo III–magnesium, explained by the size of the dye’s ionic pocket [24]. This indicates that in the presence of both ions, which are found in plasma, the probe will preferentially bind to calcium ions. This ensures high sensitivity in determining calcium ion concentration in patient plasma using this probe. Additionally, when Arsenazo III binds to calcium, a shift in the absorption maximum from 600 nm (free reagent in water) to 650 nm for the complex is observed, while the maximum for the magnesium–Arsenazo III complex lies at 620–630 nm, allowing spectroscopic differentiation of these two ions’ binding to the probe. However, to investigate whether the presence of other divalent ions could affect the absorption spectrum of the hydrogel + Arsenazo III + Ca^2+^ system at ~650 nm, we studied the absorption spectra of the hydrogel + Arsenazo III system in the presence of physiologically relevant concentrations of divalent ions found in the human body, as found in normal plasma (see Figure S9). It can be seen that under these experimental conditions (hydrogel pH, physiological concentrations of divalent ions, room temperature), the presence of other divalent ions does not affect the absorption spectrum of the fluorescent Arsenazo III probe incorporated into the hydrogel. This suggests that interference effects are negligible, and the developed platform can be considered highly selective for calcium ions in patient blood plasma.

In this work, gel parameters were empirically selected to meet several criteria: (1) The gels should not contain components that produce an absorption peak at 650 nm when interacting with the calcium probe, which is used to assess calcium content in patient plasma applied to the gel. If such components were present, they would hinder data interpretation upon plasma application. (2) The gels should have a structure allowing calcium ions from plasma to pass into the gel without changing their concentration. (3) For the task involving whole-blood application, gel pores should allow plasma to pass but not erythrocytes to avoid spectral contribution of hemoglobin.

To address these requirements, several gels were investigated: polyacrylamide (PAAG), PVA gel (formed from polyvinyl alcohol), self-assembling Fmoc-FF gel, carbomer, and gelatin gel. PAAG, PVA, and carbomer, carbopol gels were unsuitable in composition, as incorporation of Arsenazo III resulted in an absorption band at 650 nm (responsible for probe–calcium binding) even before calcium addition, hindering data interpretation. Arsenazo III, in addition to binding calcium ions, is also capable of nonspecific interactions with macromolecules, including proteins and polar functional groups of polymers, which alters its spectral characteristics [37]. In hydrogels based on PAAG, PVA, carbomer, and carbopol functional groups such as amides, hydroxyls, and carboxyls can form weak electrostatic or hydrogen bonds with Arsenazo III, resulting in the appearance of a peak around 650 nm in the absorption spectrum even in the absence of calcium (see Figure S2). For example, in [38,39], PVA has been shown to be effective as an adsorbent for heavy metal ions as well as anionic and cationic dyes, owing to the large number of free hydroxyl (–OH) and acetate (–O–CO–CH_3_) groups present along its polymer chains. Binaeian et al. demonstrated that PAAG hydrogel is able to bind anionic dyes via hydrogen bond formation (Dye–NH_3+_), a class to which Arsenazo III also belongs [40]. The self-assembling Fmoc-FF hydrogel is unsuitable as it is a soft gel (Young’s modulus E = 400–600 Pa [33]), with loosely arranged fibrils, allowing not only plasma but also erythrocytes to easily pass through, thus not meeting our criteria (Figure S3).

As a result, gelatin gel was found to satisfy all requirements. The gel composition was selected empirically, with peptide and probe concentrations varied in the experiment.

Initially, calcium solution at concentrations typical for healthy donor plasma was applied to the gelatin hydrogel. After diffusion into the hydrogel, the Arsenazo III spectrum (with a maximum at 535 nm; see Figure 2A, pink curve) developed three absorption peaks: a red shift in the Arsenazo peak to λ = 545 nm and two new peaks at λ = 600 nm and λ = 650 nm (see blue curves, Figure 2A), corresponding to calcium–Arsenazo III complex formation. The simultaneous increase in these two peaks upon sequential calcium addition confirms that they represent two electronic transitions in the same molecule (see Figure S4). Similar spectrum changes were observed upon addition of lanthanide ions to the probe solution [41,42].

Experiments showed a linear dependence of Arsenazo III optical density at 650 nm on added calcium concentration in solution applied to the hydrogel.

Further, the hydrogel + Arsenazo III system was tested for precise determination of plasma calcium concentration. First, donor plasma calcium concentration was determined using the clinical “calcium-Olveks” reagent. Then, plasma was applied to the hydrogel, and calcium concentration passing into the gel + Arsenazo III system was determined using Formula (3), with a known-concentration calcium solution (2.3 mM) as calibrator. Gelatin concentration was varied in these experiments. Table 1 presents a comparison of calcium concentrations from different donors as assessed by clinical analysis and the hydrogel.

Table 1 shows that for gels with gelatin content in the range 6–9%, calcium concentration results from both methods were in good agreement. For gels with 12–18% gelatin, calcium concentration in the gel differed slightly from that in the clinical analysis results. The largest discrepancy was observed for the 5% gelatin gel: calcium concentration in the gel was 1.42 mM, while clinical analysis gave 2.16 mM. Such differences were due to the low gelatin concentration resulting in a very soft gel that poorly retained its structure upon plasma application. Overall, it can be concluded that the maximum deviation of the obtained data from the clinical values was 0.06 mM, and the normal range of calcium concentration in blood plasma is 2.15–2.50 mM. Such deviations from the clinical values allow the use of this method for measuring the clinically relevant Ca^2+^ fraction in the human body.

The ratio of calcium to Arsenazo III concentration also varied. It was shown that plasma penetrated the hydrogel without changing calcium concentration for *C*_CaCl2_/*C*_ArsenazoIII_ = 10–40. At higher ratios, the amount of calcium–Arsenazo III complexes formed was too small, and Arsenazo III optical density at 650 nm in the gel was almost insensitive to their presence (see Figure 3D,E).

Additionally, an experiment was conducted to determine whether these gels can be used to measure calcium ion concentration in whole blood. In the experiments utilizing the gel as a platform for whole-blood separation, a drop of whole blood (50 μL) was placed on the surface of the gel and incubated for 10 min, after which it was washed off with 5 mL of distilled water. After the removal of erythrocytes, calcium that had passed with the plasma into the hydrogel was measured spectrophotometrically. The gel was required to possess a structure such that the pore size allowed free passage of calcium ions and calcium-bound plasma proteins into the gel, while all erythrocytes, which were characterized by a size of 7–10 μm, remained on the surface of the hydrogel and were subsequently removed prior to measurement.

A calibrator (2.3 mM calcium), plasma, and whole blood from the same donor were applied to the gels, and calcium concentrations passing into the hydrogel + Arsenazo III system were compared. The results are shown in Table 2.

It is evident that only for 7% and 8% gels were all three concentration values close. With increased peptide concentration, pore size decreased, explaining why plasma and its components penetrated the gel less effectively at higher gelatin concentrations. Scanning ion conductance microscopy showed that pore sizes in gelatin gels at 7% and 8% concentrations were 300–600 nm.

Finally, it should be noted that the developed method has both advantages and disadvantages. A comparison of the proposed method with ion-selective electrode-based PoC devices and microfluidic chips—widely used point-of-care methods for Ca^2+^ concentration determining in blood plasma—can be seen below (Table 3).

The proposed method allows rapid and accurate determination of ionized calcium concentration in blood with minimal sample preparation: a single drop of whole blood is sufficient. The analysis takes only 10–15 min and can be performed using readily available equipment, without the need for specialized ISE analyzers or complex microchips. Optimization of gelatin and sensor concentrations ensures reproducibility and accuracy across different samples, making the method suitable for PoC applications. Thus, the method combines simplicity, speed, and low cost, distinguishing it from existing calcium analysis technologies. Moreover, the measures ensure the robustness, reproducibility, and practical applicability of the assay for point-of-care calcium determination in blood samples; please see the section “Standardization of the Method and Robustness Testing”.

As shown in the table, compared to ISE-PoC methods, the main advantage of our approach is the ability to use fingerprick blood samples, whereas ISE-PoC methods predominantly require venous blood. Another advantage is the small sample volume required (from 10 μL), which is particularly important for pediatric and critically ill patients.

## 3. Conclusions

In this study, a rapid point-of-care method for determining calcium concentration in plasma and whole blood using gelatin hydrogel modified with Arsenazo III was developed and tested. Experimentally, it was established that gelatin hydrogel with *C*_gelatin_ = 6–18% and *C*_arsenazoIII_ = 6 × 10^−5^–2 × 10^−4^ M provides high measurement accuracy, allowing detection of hypocalcemia or hypercalcemia, i.e., monitoring calcium metabolism pathologies. For whole-blood analysis, the optimal gelatin concentration range is 7–8%, at which plasma with calcium efficiently diffuses into the gel while erythrocytes remain on the surface. The method offers several advantages, making it promising for point-of-care use: rapid analysis, simple sample preparation, and applicability to both plasma and whole blood. Limitations include the need for calibration and precise gel parameter selection for whole-blood analysis. Overall, the proposed technology can significantly simplify and accelerate the determination of calcium concentration in biological fluids and improve diagnostic efficiency in point-of-care settings.

## 4. Materials and Methods

### 4.1. Sample Preparation

(A) Reagents. Bovine skin gelatin, phosphate-buffered saline (PBS), peptide N-fluorenylmethoxycarbonyl-diphenylalanine (Fmoc-FF), acrylamide, N,N′-methylenebis (acrylamide) (bis-acrylamide), glutaraldehyde, ammonium persulfate, polyacrylate, and allyl pentaerythritol were purchased from Sigma-Aldrich (St. Louis, MO, USA). TEMED was purchased from Fluka (Darmstadt, Germany). The calcium probe Arsenazo III and calcium chloride were obtained from Khimreaktivy (St. Petersburg, Russia), and calcium-olweks was obtained from Olvex Diagnosticum (Moscow, Russia).
(B) Hydrogels. In the first stage, the formation of different gels and their interaction with the calcium probe Arsenazo III were studied. The following gels were considered: (1) polyacrylamide gel (PAAG), (2) PVA gel (formed from polyvinyl alcohol), (3) self-assembling Fmoc-FF peptide hydrogel, (4) carbomer gel, (5) carbopol gel, and (6) gelatin gel. As the platform for calcium determination in patient blood plasma, a gel made from bovine skin gelatin was selected; all other gels were unsuitable for this purpose. Preparation methods are given in the Supplementary Information. Gels with various gelatin concentrations were used: *C*_gel_ = 5–18%. The required mass of gelatin was mixed with mQ water, heated to t = 50 °C, and stirred with a magnetic stirrer. After thorough mixing, the calcium probe Arsenazo III was added, and 120 μL of gel was poured into Petri dishes and allowed to cool at room temperature for several hours to form the gel. The concentration of Arsenazo III probe was varied in the range *C*_ars_ = 3 × 10^−5^ M–2.5 × 10^−4^ M. The pH of the samples was controlled, as a mildly acidic environment (pH of 6–7) is required for selective binding of Arsenazo III to calcium. The pH of hydrogels with Arsenazo III in experiments was 6.5. Test samples (CaCl_2_ as calibrator, blood plasma, or whole blood) were applied to the prepared gel in 50 μL volumes and allowed to diffuse into the gel for 10 min; the residual drop (not absorbed into the gel) was removed, and then absorption spectra of the sample that entered the gel were measured. The thickness of hydrogels was 0.1 cm.

### 4.2. Blood Collection and Application to Hydrogel

The study was conducted in accordance with the Declaration of Helsinki under a protocol approved by the Ethics Committee of CTF FHP RAS on 17 January 2023 (permit number: 1/2–23(HЭK)). All participants provided informed consent. The study included 20 healthy volunteers not taking any medications. Blood was collected into tubes containing sodium heparin (4.5 mL). For the experiment, 50 μL of (1) blood plasma or (2) whole blood was applied to the hydrogel, with gel parameters (gelatin and Arsenazo III concentrations) selected so that plasma with a calcium concentration matching the calibrator would penetrate the gel, which was also applied as a reference. Calcium chloride with a concentration *C*_CaCl2_ = 2.3 mM was used as the calibrator, corresponding to the calcium ion concentration in healthy human plasma (*C*_CaCl2_ = 2.15–2.5 mM). Calcium concentration in blood samples was controlled using calcium-olweks (Olvex Diagnosticum, Moscow, Russia), a reagent kit for quantitative determination of calcium in human blood by colorimetry in clinical laboratory diagnostics. After 10 min, any sample remaining on the gel surface was removed, and the absorption spectrum of the sample that entered the gel was measured.

### 4.3. Determination of Calcium Ion Concentration by Absorption Spectroscopy

Absorption spectra were measured using a Lambda 25 spectrophotometer (Perkin Elmer, Waltham, MA, USA). The absorption spectra of Arsenazo III embedded in gelatin hydrogel (*λ*_max_ = 650 nm) and the Arsenazo III + calcium chloride (CaCl_2_) system (from calibrator or blood plasma) were measured in the range 400–750 nm. Measurements were performed in a Petri dish; gel layer thickness was about 1 mm. The optical density D of the samples was measured, determined by the following formula:

(2) D=c·l·ε

where *c* is the concentration of the substance, *l* is the optical path length, and *ε* is the molar extinction coefficient. The wavelength range studied was 400–750 nm. The optical path length was equal to 0.1 cm. Each sample was measured 3 times. The standard deviation of measured data was about 5%. All graphs were created using OriginPro 2015 software.

### 4.4. Determination of Hydrogel Pore Diameter by Scanning Ion Conductance Microscopy

The topography of the gelatin hydrogel was studied using a scanning ion conductance microscope (SICM) (ICAPPIC Limited, London, UK). An inverted optical microscope Olympus IX73 (Toyko, Japan) served as the SICM platform. Feedback control and piezoelement positioning were performed using the ICAPPIC Universal Controller and Piezo Control System (ICAPPIC Limited, UK). Borosilicate glass pipettes with an internal radius of 45–60 nm were fabricated using a P-2000 laser puller (Sutter Instruments, Novato, CA, USA) for topography studies. The exact internal radius was calculated by the following formula [43]:

(3) I=π·r·k·V·tan (α)

where *I* is the ionic current, α is the half-cone angle (3 degrees), *k* = 1.35 cm·m^−1^, and *V* is the applied voltage (200 mV). Ionic current values ranged from 2000 to 2600 pA, corresponding to capillary radii of 45–60 nm. Hydrogel topography was studied by the non-contact hopping mode [44] with an ion current drop of 0.5%, scan size of 6 × 6 μm, and resolution of 256 × 256 pixels. The current drop rate during topography measurement was 120 μm·s^−1^.

## Figures and Tables

**Table 1:** Comparison of calcium concentration values in donor blood plasma assessed by calcium-Olvex (colorimetric method, in solution) and for plasma applied to hydrogel, in hydrogels with different gelatin concentrations. *C*_arsenazoIII_ = const = 7 × 10^−5^ M.

Gelatin Concentration in Hydrogels (%)	Calcium Concentration in the Blood Plasma of Different Donors, Assessed by Clinical Analysis, (mM)	Calcium Concentration in the Blood Plasma of Different Donors After Adhesion of Plasma on the Hydrogel (mM)
5	2.16	1.42
6	2.16	2.17
7	2.20	2.22
9	1.96	1.95
12	2.22	2.17
18	2.24	2.30

**Table 2:** Comparison of calcium concentration values in donor blood plasma assessed by calcium-Olvex (colorimetric method, in solution), for plasma applied to hydrogel and for whole blood applied to hydrogel, with different gelatin concentrations. *C*_arsenazoIII_ = const = 7 × 10^−5^ M.

Gelatin Concentration in Hydrogels (%)	Calcium Concentration in the Blood Plasma of Different Donors, Assessed by Clinical Analysis (mM)	Calcium Concentration in the Blood Plasma of Different Donors after Adhesion of Plasma on the Hydrogel (mM)	Calcium Concentration in the Blood Plasma of Different Donors After Adhesion of Whole Blood on the Hydrogel (mM)
5	2.16	1.42	1.42
6	2.16	2.17	1.50
7	2.20	2.22	2.15
8	2.14	2.17	2.12
9	1.96	1.95	1.6
12	2.22	2.17	1.05
18	2.24	2.3	1.3

**Table 3:** A comparison of the proposed method with ion-selective electrode-based PoC devices and microfluidic chips.

	ISE-PoC	Microfluidic Chips	Hydrogel Platform
Sample type	Whole blood, plasma	Plasma	Whole blood, plasma
Form of Ca detected	Ionized Ca^2+^	Total or ionized Ca^2+^	Ionized Ca^2+^
Equipment required	Specialized analyzer	Microfluidic system	Photometric system
Assay time	1–3 min	5–15 min	10–15 min
Limitations	Requires calibration	Requires calibration, complex fabrication, possible chip clogging, high equipment cost	Requires calibration
Blood collection	Venous blood (sometimes capillary blood)	Venous blood (sometimes capillary blood)	Capillary blood
Accuracy	Measures clinically relevant Ca^2+^ fraction	High correlation with standard lab methods	Measures clinically relevant Ca^2+^ fraction
Sample volume	60–100 μL	10–50 μL	10–50 μL

## Data Availability

No new data were created or analyzed in this study.

## Supplementary Materials

The following supporting information can be downloaded at https://www.mdpi.com/article/10.3390/gels12010028/s1. Figure S1: Photographs of (A) a gelatin hydrogel incorporating the calcium probe Arsenazo III, (B) a drop of blood plasma applied to the hydrogel under study, (C) the hydrogel after removal of the plasma drop following 10 min of sample incubation on the hydrogel (i.e., the form in which the sample would be analyzed photometrically), (D) a drop of whole blood applied to the hydrogel under study, and (E) the hydrogel after removal of the whole blood drop following 10 min of incubation and rinsing with 5 mL of distilled water (i.e., the form in which the sample would be analyzed photometrically). Figure S2: Absorption spectra of the PAAG + Arsenazo III, PVA + Arsenazo III, and (C) Carbomer TC 340 + Arsenazo III systems in the absence of calcium ions. Figure S3: Absorption spectra of the Fmoc-FF + Arsenazo III + blood, gelatin (6%) + Arsenazo III + blood, and gelatin (18%) + Arsenazo III + blood systems. On the left, photographs show a drop of blood applied to the gels, which was removed from the gel surface after 10 min. Figure S4: Normalized optical density values of the peaks for the Arsenazo III + CaCl_2_ system in gelatin hydrogel at wavelengths of 650, 600, and 560 nm. Figure S5. Absorption spectra of Arsenazo III probe added to Sigma-Aldrich gelatin gel, G9382 (black line), Sigma-Aldrich gelatin gel, G9391 (red line), food-grade gelatin #1 (blue line), and food-grade gelatin #2 (pink line). Figure S6. Absorption spectra of the gelatin gel + Arsenazo III system after application of patient plasma following thawing for 5 min (red line), 10 min (green line), and 20 min (blue line). Figure S7. Absorption spectra of the gelatin gel + Arsenazo III system after application of patient plasma following thawing after 1 day (black line), 3 days (red line), and 7 days (blue line) of storage. Figure S8. Absorption spectra of the gelatin gel + Arsenazo III system after application of whole blood and subsequent washing with 5 mL (black line), 10 mL (red line), and 15 mL (blue line) of distilled water. For comparison, the absorption spectrum after application of plasma from the same patient is also shown. Figure S9. Absorption spectrum of pure Arsenazo III in gelatin hydrogel (black curve) and after addition of calcium ions (red curve), magnesium ions (blue curve), cooper ions (pink curve) and zinc ions (green curve). The concentrations of divalent ions correspond to their concentration in normal in blood plasma: *C*_Ca2+_ = 2.3 mM, *C*_Mg2+_ = 1 mM, *C*_Cu2+_ = 17 mkM, and *C*_Zn2+_ = 14 mkM. *C*_Arsenazo III_ = cons t= 70 mkM [45,46,47].

## Abbreviations

The following abbreviations are used in this manuscript:

PVA	Polyvinyl acetate
PAAG	Polyacrylamide gel
Fmoc-FF	Fluorenylmethoxycarbonyl-diphenylalanine
