# Supplementary material for: Hydrogel as a Platform for Point-of-Care Calcium Determination in Blood
Source: Gels. 2025 Dec 29;12(1):28. doi: 10.3390/gels12010028 (PMC12840776; doi:10.3390/gels12010028)
Supplement: Supplementary file 1 [file gels-12-00028-s001.zip › gels-4038459-supplementary.pdf]

Supporting Information

Hydrogel as a platform for point-of-care calcium determination in blood

Tatiana N. Tikhonova [1]*, Anastasia V. Barkovaya [1], Yuri M. Efremov [2], Vladimir I. Panov[1], Peter S. Timashev [2,3,4], Victor V. Fadeev [1]

[1] Department of Physics, M.V. Lomonosov Moscow State University, Leninskie gory 1/2, 119991, Moscow, Russia; tikhonova@physics.msu.ru, anastasia.bark18@gmail.com, panov@spmlab.phys.msu.ru, vfadeev@physics.msu.ru

[2] Institute for Regenerative Medicine, Sechenov University, 8-2 Trubetskaya st., 119991, Moscow, Russia; yu.efremov@gmail.com

[3] Department of Chemistry, M.V. Lomonosov Moscow State University, Leninskie gory 1/2, 119991, Moscow, Russia; timashev.peter@gmail.com

[4] World-Class Research Center "Digital biodesign and personalized healthcare", Sechenov First Moscow State Medical University 8-2, Trubetskaya st., 119991, Moscow, Russia.

* Correspondence: tikhonova@physics.msu.ru; +7-495-939-12-25

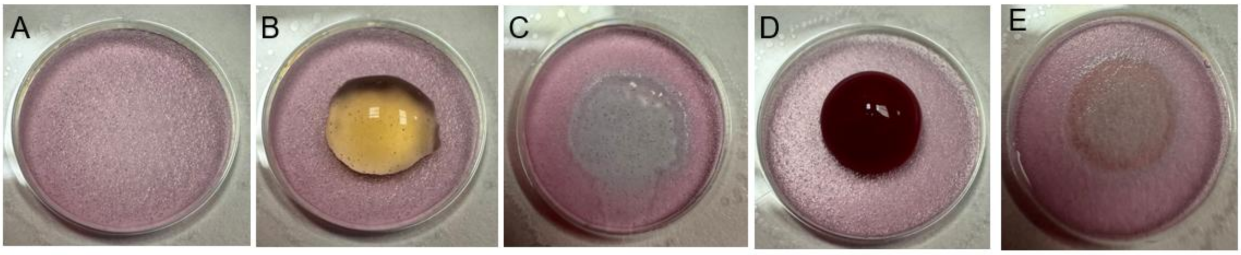

Figure S1. Photographs of (A) a gelatin hydrogel incorporating the calcium probe arsenazo III, (B) a drop of blood plasma applied to the hydrogel under study, (C) the hydrogel after removal of the plasma drop following 10 minutes of sample incubation on the hydrogel (i.e., this is the form in which the sample will be analyzed photometrically), (D) a drop of whole blood applied to the hydrogel under study, and (E) the hydrogel after removal of the whole blood drop following 10 minutes of incubation and rinsing with 5 mL of distilled water (i.e., this is the form in which the sample will be analyzed photometrically).

Parameters of the samples under study: gelatin gel concentration $C_{gelatin}$ = 7%, arsenazo III concentration $C_{arsenazoIII} = 6.5 \times 10^{-5}$ M. The gel thickness is 0.1 cm.

Methods for Hydrogel Preparation

1. **Polyacrylamide Gel (PAAG).** Polyacrylamide hydrogel was prepared according to a well-established protocol [Tse]: C_{acryl} = 4%, $C_{bis\text{-}acryl}$ = 0.1%. Aqueous solutions of acrylamide and bis-acrylamide in Milli-Q water were polymerized with 0.1% initiator (ammonium persulfate) and 0.1% catalyst (TEMED) between two square glass coverslips (Menzel–Gläser, Germany).
2. **Self-assembling Peptide Gel Fmoc-FF.** The Fmoc-FF hydrogel was prepared using the solvent-switch method: peptides were dissolved in dimethyl sulfoxide (DMSO) to obtain a stock solution, $C_{Fmoc\text{-}FF}$ (stock) = 10%, and then the stock solution was diluted in mQ-water to a final concentration of $C_{Fmoc\text{-}FF}$ = 0.6%. After hydrogel formation, the gels were thoroughly washed with PBS buffer solution for 2 days to ensure complete removal of DMSO [Kolenc] (for the solvent-switch method), and to adjust the hydrogel pH to 7.3 instead of pH 4.
3. **PVA Gel (formed from polyvinyl alcohol).** A 5% PVA solution was prepared by dissolving PVA powder in mQ-water with magnetic stirring for 2 hours in a water bath at 85°C. The gels were then formed by cooling to room temperature [Fan].
4. **Carbomer TC 340 and Carbopol NF 2020 Gel.** Gels were prepared using the same procedure. Polymers were used at concentrations of 1%, 0.5%, 0.25%, and 0.1%. The required amount of polymer was gradually added to water with continuous stirring. The solution was stirred for 10 minutes, after which 18% NaOH solution was added to adjust the gel to pH 7.0. Upon addition, the solution immediately formed a gel.

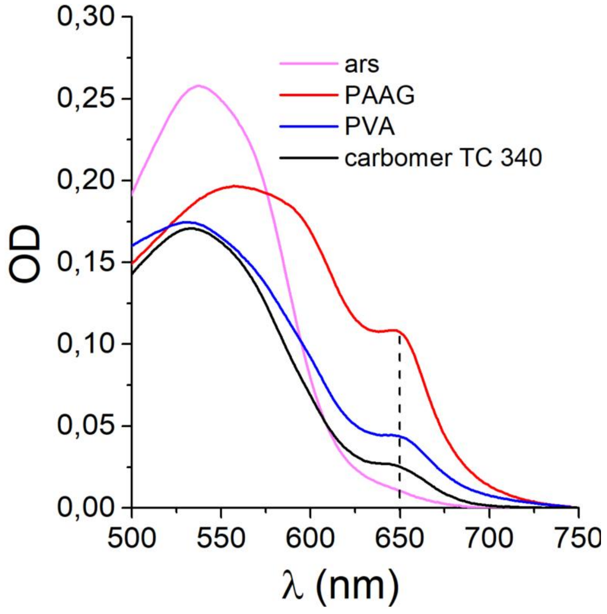

Figure S2. Absorption spectra of the PAAG+Arsenazo III, PVA+Arsenazo III, and (C) Carbomer TC 340+Arsenazo III systems in the absence of calcium ions.

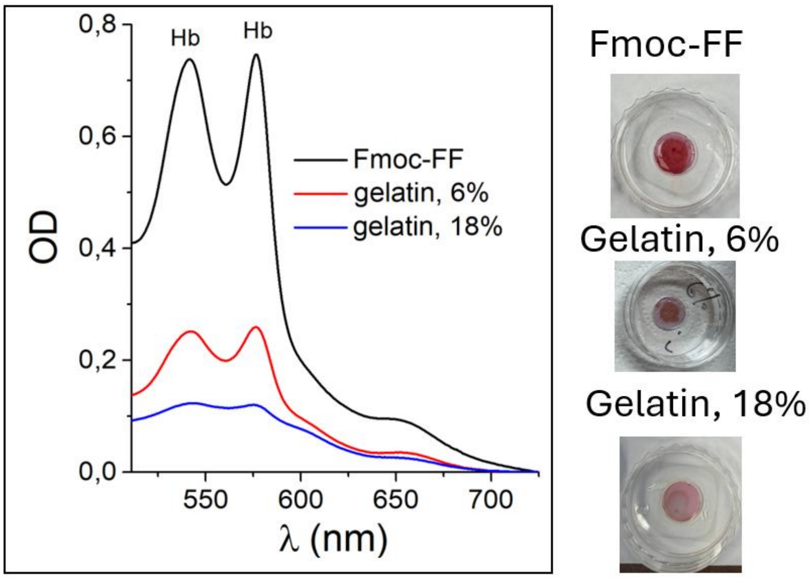

Figure S3. Absorption spectra of the Fmoc-FF+Arsenazo III+blood, gelatin (6%)+Arsenazo III+blood, and gelatin (18%)+Arsenazo III+blood systems. On the left, photographs show a drop of blood applied to the gels, which was removed from the gel surface after 10 minutes.

The photographs demonstrate that the blood drop spread on the Fmoc-FF gel and a much higher concentration of hemoglobin penetrated into the Fmoc-FF gel compared to the gelatin gels with 6% and 18% gelatin concentrations, respectively. This effect is reflected in the absorption spectra of the studied systems. It is evident that the hemoglobin peaks at 540 nm and 575 nm for the Fmoc-FF+Arsenazo III+blood system have a shoulder in the long-wavelength region, which may interfere with data interpretation for the peak at 650 nm.

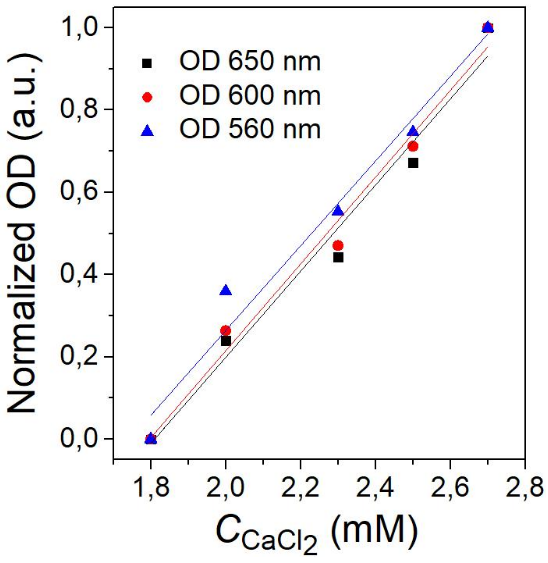

Figure S4. Normalized optical density values of the peaks for the Arsenazo III+$CaCl_2$ system in gelatin hydrogel at wavelengths of 650, 600, and 560 nm.

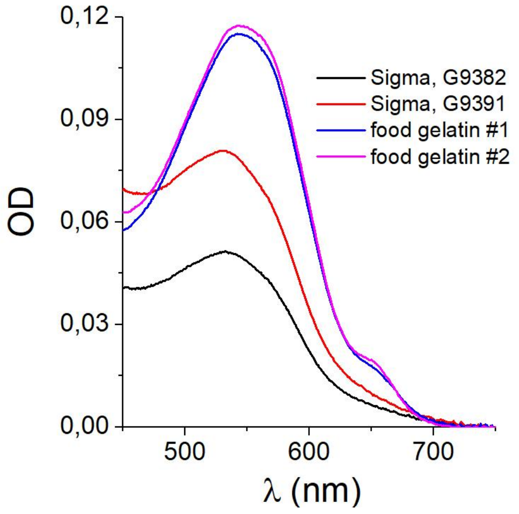

Fig. S5. Absorption spectra of Arsenazo III probe added to Sigma-Aldrich gelatin gel, G9382 (black line), Sigma-Aldrich gelatin gel, G9391 (red line), food-grade gelatin #1 (blue line), food-grade gelatin #2 (pink line).

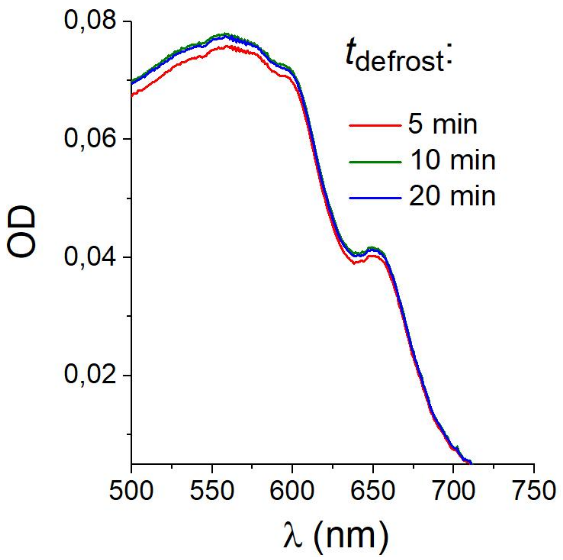

Fig. S6. Absorption spectra of the gelatin gel+Arsenazo III system after application of patient plasma following thawing for 5 min (red line), 10 min (green line), and 20 min (blue line).

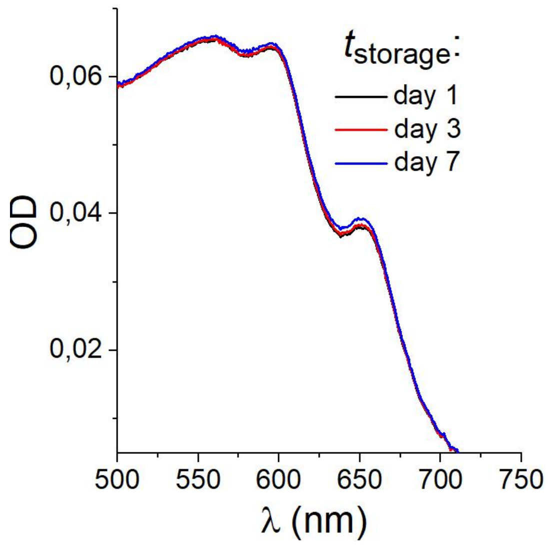

Fig. S7. Absorption spectra of the gelatin gel+Arsenazo III system after application of patient plasma following thawing after 1 day (black line), 3 days (red line), and 7 days (blue line) of storage.

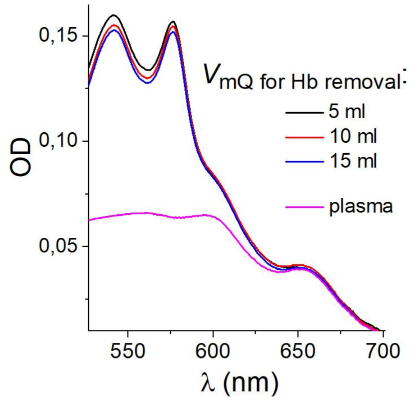

Fig. S8. Absorption spectra of the gelatin gel+Arsenazo III system after application of whole blood and subsequent washing with 5 mL (black line), 10 mL (red line), and 15 mL (blue line) of distilled water. For comparison, the absorption spectrum after application of plasma from the same patient is also shown.

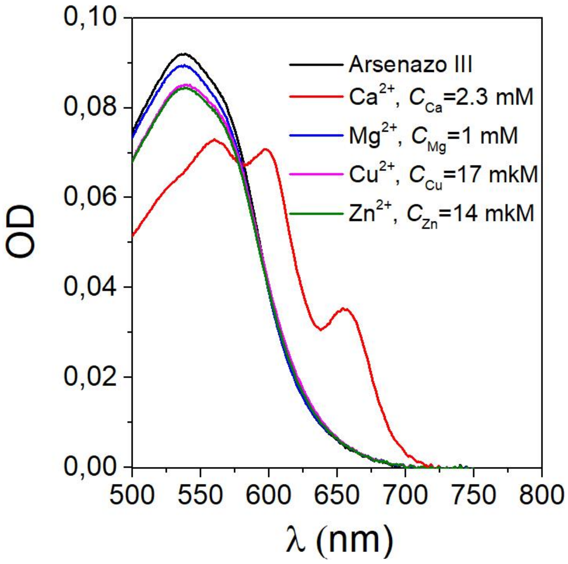

Figure S9. Absorption spectrum of pure arsenazo III in gelatin hydrogel (black curve) and after addition of calcium ions (red curve), magnesium ions (blue curve), cooper ions (pink curve) and zinc ions (green curve). The concentrations of divalent ions correspond to their concentration in normal in blood plasma: C_{Ca2+}=2.3 mM, C_{Mg2+}=1 mM, C_{Cu2+}=17 mkM, C_{Zn2+}=14 mkM. $C_{Arsenazo\ III}$=const=70 mkM.

References:

- O.I. Kolenc, K.P. Quinn. Antioxid. Redox Signal., 30 (6), 875 (2019). DOI: 10.1089/ars.2017.7451

- J. R. Tse, A. J. Engler, *Curr. Protoc. Cell Biol.*, 2010, 47, 10.

- Fan, L., Yang, H., Yang, J., Peng, M., & Hu, J. (2016). Preparation and characterization of chitosan/gelatin/PVA hydrogel for wound dressings. *Carbohydrate polymers*, *146*, 427-434.
